# P-688. PFKFB3-driven Glycolysis Controls Influenza A Virus Entry

**DOI:** 10.1093/ofid/ofaf695.901

**Published:** 2026-01-11

**Authors:** Ronghui Liang

**Affiliations:** UNIVERSITY OF HONG KONG, HONGKONG, Not Applicable, Hong Kong

## Abstract

**Background:**

Novel therapeutic intervention in acute respiratory virus infections remains a critical challenge due to high viral burden and severe inflammation. Glucose metabolism is a central driver of viral replication and host immune responses. The host enzyme 6-phosphofructo-2-kinase/fructose-2,6-bisphosphatase 3 (PFKFB3) regulates glycolytic flux and may represent a metabolic checkpoint during infection.

**Methods:**

Using influenza A H1N1 virus, we employed viral-host interaction assays, and real-time glycolysis monitoring (extracellular acidification rate analysis) to evaluate virus-induced metabolic reprogramming. Antiviral assays targeting PFKFB3 were conducted using genetic editing and pharmacological inhibition *in vitro*. A conditional lung-specific knockout mouse model (C57BL/6JCya-*Pfkfb3^em1flox^*/Cya) was generated to examine *in vivo* relevance.

**Results:**

PFKFB3 expression was significantly upregulated in H1N1-infected BEAS-2B cells. Among viral proteins screened, PB1, PB2, PA, NS, and NA enhanced basal glycolysis, with PB1 inducing a dose-dependent increase in ECAR. Confocal microscopy revealed robust PB1–PFKFB3 colocalization. PFKFB3 deletion or inhibition reduced viral replication *in vitro* and in the conditional knockout mouse model. Mechanistically, PFKFB3 activity promoted glycosylation of host viral entry receptors, facilitating viral entry.

**Conclusion:**

PFKFB3 acts as a host-encoded proviral factor during influenza A infection by enhancing glycolysis and receptor glycosylation. Targeting PFKFB3 offers a promising antiviral strategy in both cellular and animal models.

**Disclosures:**

All Authors: No reported disclosures

